# Gameplay and physical activity behaviors in adult video game players

**DOI:** 10.3389/fspor.2024.1520202

**Published:** 2025-01-06

**Authors:** Haylei Scoggins, Ryan R. Porter, Robyn Braun-Trocchio

**Affiliations:** Department of Kinesiology, Texas Christian University, Fort Worth, TX, United States

**Keywords:** exercise, sedentary behavior, physical health, video game genres, adult gamers

## Abstract

**Introduction:**

Since the early 2000s, the video game industry has seen extraordinary booms in product development and market growth, with the total number of video game players globally reaching 2.69 billion by the end of 2020. Despite the rapid growth of the industry, there is little recent data investigating the time adult video game players spend sedentary playing video games and the time they spent engaged in physical activity. The purpose of this quantitative, cross-sectional, non-experimental survey study is to describe the frequency and duration of video game play and physical activity in adult video game players.

**Methods:**

Participants completed an online survey, evaluating their demographic and health history information, video game play, and physical activity behaviors.

**Results:**

The study used data from a total of 221 participants (Males = 153, Females = 68). The mean age of the participants was 27.29 (SD 7.27) years. Of the 221 participants, 145 identified as casual players, 50 amateurs, 24 semi-professionals, and 2 professionals. The participants spent over five days per week and an average of 26.56 h per week playing video games. Personal computers were reported to have the longest duration of play of the four platforms investigated (17.59 h per week). The total amount of time participants spent engaged in cumulative moderate-to-vigorous physical activity (MVPA) was an average of 15.43 (SD 16.79) hours per week. The majority of this time was spent engaged in occupational physical activity (5.11 h per week). Participants spent 2.39 h per week engaged in leisure time MVPA.

**Conclusion:**

Our results indicate an increase in VG play compared to 2018, suggesting United States adult video game players may be more at risk for detrimental effects to their physical health. This could be attributed to the habits formed during the COVID-19 pandemic, the influences from video genre game play mechanics, and the social aspects of playing video games with friends. Future research should focus on developing research methodologies that will objectively measure adult video game player frequencies and durations in video game play alongside extensive observation of different video gameplay mechanic genres and their relationships with physical activity.

## Introduction

Since the early 2000s, the video game (VG) industry has seen extraordinary growths in product development and market expansion. From 2017 to 2023 alone, the global VG market revenue increased by 223.53 Billion United States Dollars [BUSD]) and is expected to increase by an additional 136.82 BUSD by 2027 (1). This revenue has surpassed both the projected total global box office for the film industry in 2023 ($30 BUSD) (2) and the global music industry in 2022 ($26.2 BUSD) (3). The combined revenue of both the film and music industry totals does not even come close to the total revenue generated by the VG industry. This market growth represents the increases in casual and professional esports VG play as well as the growth in esports spectatorship. In addition to market growth, contracted professional esports play and live-streaming VGs are becoming widely accepted career choices, and universities are offering scholarships to esports athletes (4, 5).

Research has shown associations between physical inactivity, prolonged sedentary time, and increased screen time with elevated risk for developing chronic health conditions (CHCs) such as obesity, diabetes, musculoskeletal pains, cardiovascular disease (CVD), or overall mortality (6–9). More specifically, focusing on esports athletes, results have demonstrated that this group is not only at risk for these CHCs, but also other risks such as muscle weakness, vision fatigue, poor sleep, and nutritional deficits (10–14). It is well known that prolonged sedentary time, including VG play, puts millions at risk of developing such CHCs or experiencing premature mortality (6–9, 12). Adult video game players (VGPs) have a higher likelihood of developing CHCs due to the sedentary nature of VG play compounding with other daily sedentary behaviors (e.g., work- or school-based activities, household, and transportation), putting them at risk of early mortality. Regardless of player skill level, the danger of developing CHCs leading to premature mortality in this population is a potential consequence of prolonged VG play. In a study observing twenty-four cases of mortality associated with prolonged sedentary VG play between 2002 and 2021, nineteen were adults aged between 18 and 40 (15). All fatalities were associated with VG play, with four of the deceased adult victims reported to have co-morbidities including obesity, asthma, previous heart attack or CVD, high blood pressure, or liver disease (15).

Despite this knowledge, adult VGPs spend long periods daily playing VGs on top of their other daily sedentary behaviors. In 2009, Ballard et al. reported an average of 63.36 min per day spent playing VGs (16), while in 2018, Arnaez et al. found an average of 2.75, 2.25, and 1.25 h per day spent playing personal computer (PC), console, and other electronic games respectively (17). Due to the restrictions caused by the COVID-19 pandemic, VG play times were exacerbated, with one study finding a 74.6% increase in gaming time amongst casual and competitive adult VGPs (18). These increases in gaming time may further exacerbate the likelihood of developing physical health detriments. As previously stated, this VG play time is compounded on top of other daily sedentary behaviors, in which studies have found a significant increase in non-VG specific sedentary behavior compared between pre- and post-pandemic restrictions (19, 20). Such increases in non-VG specific sedentary behavior further exacerbates the likelihood of this population developing CHCs.

To prevent, mitigate, and reduce the risks of developing CHCs and avert premature mortality, guidelines from the World Health Organization (WHO) and the United States (U.S.) Centers for Disease Control (CDC) recommend adults spend 150–300 min per week engaged in moderate-to-vigorous physical activity (MVPA) (6, 9, 21, 22). However, approximately 25% of U.S. adults are sedentary and 76% of U.S. adults play VGs on at least one platform (23–25). During the pandemic, there was a 24.7% decrease in physical activity (PA) and a 64.3% increase in sitting behavior among adult VGPs (18). A meta-analysis conducted by Chau et al. (2013) identified that sitting for eight or more hours per day was associated with a significantly higher risk of mortality (8). Due to the changes in work and PA habits from the pandemic, it is likely adult VGPs are either meeting or surpassing eight hours of sedentary time per day.

Unfortunately, little research has encompassed all adult VGPs and their physical health, much less how long they spend playing VGs. To date, the majority of research investigating the relationship between VG play and physical health typically has solely child and adolescent populations. This research, in conjunction with research on other screen time use, has led to the creation of screen time guidelines for children and adolescents such as those provided by the American Academy of Pediatrics (26). In contrast, the dearth of evidence pertaining to adult sedentary behavior has resulted in the inability to create quantified guidelines and recommendations for this population (27). Adults at high risk of cumulating extensive hours of sedentary behavior over the course of a week, such as those with sedentary occupations and hobbies (e.g., adult VGPs), are facing an increased likelihood of developing the previously mentioned CHCs. To counteract these problems, researchers and practitioners need to develop appropriate, updated interventions and guidelines targeting high-risk adults. Achieving this goal starts with obtaining updated, current measurements of how long adults are spending sedentary playing VGs. To achieve this goal, the purpose of this study was to describe the characteristics of physical activity and VG play behaviors in adult VGPs.

## Methods and materials

### Participants

Data was collected from adult VGPs in the U.S. A VGP was defined as someone who plays VGs regardless of consistent frequency or duration of engagement. See the Design and Instrumentation and Procedure sections for detailed information on how participants were recruited and how the data were collected.

**Figure 1:**
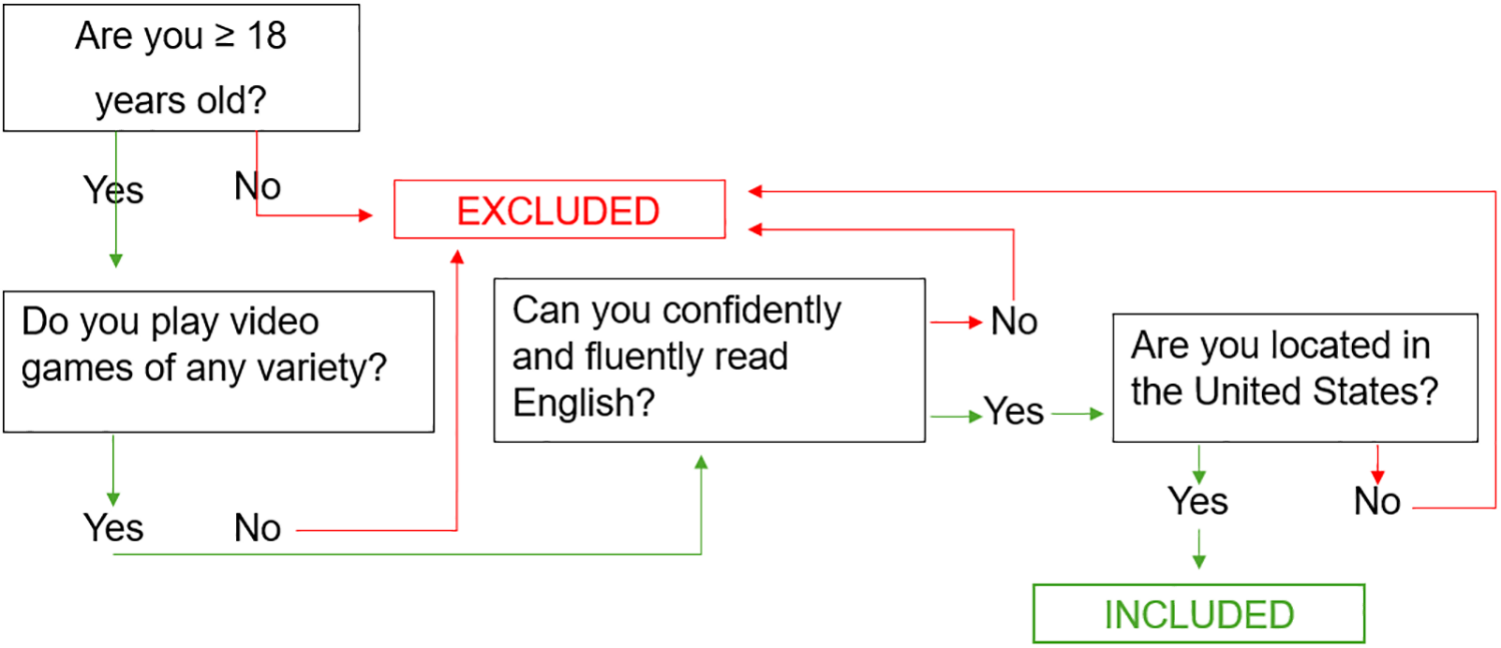
Flowchart of participant inclusion criteria.

Of the total 811 participants invited to complete the survey 259 completed it in its entirety, resulting in a 31.9% completion rate. Eligible participants comprised English speaking adults (18 years of age and older) of any sex or gender residing in the U.S. who play video games of any type on any platform. No further inclusion or exclusion criteria were implemented. See Figure 1 for a flowchart of inclusion and exclusion criteria.

Twenty-nine respondents were excluded due to inconsistent or incomplete responses, such as providing inaccurate responses, failing to follow survey instructions, or completing the survey in less than 10 min. Of the 29 excluded respondents, five participants were removed from analysis due to their responses being written in a foreign language, and 14 were removed for excessive weekly cumulative activity times (>10,080 min). Nine participants were excluded whose PA per week (post-time adjustments above) exceeded 10,080 min (168 h) per week. Outliers per VG play behavior and PA domain included participants who answered >12 h per day of VG play and or ≥3 h per day of any domain of vigorous PA. The calculations for the total amounts of time per week of VG behavior and PA domain were adjusted in SPSS to accommodate for these outliers. The total participants used in data analysis was 221. Observing binary sex, 69.2% (*N* = 153) were male and 30.8% were female (*N* = 68). Of the group, 10 identified themselves as non-binary, 3 genderfluid, and 1 as other. The mean age of the participants was 27.29 (SD 7.27) years, with the oldest participant being 62 and the youngest at 18. Of the 221, 145 were casual players, 50 amateurs, 24 semi-professionals, and 2 professionals. For additional demographic descriptive information, see Table 1.

**Table 1:** Participant demographic descriptives.

	Total	Male	Female
Sex	221 (100%)	153 (69.2%)	68 (30.7%)
	Mean (SD)	Mean (SD)	Mean (SD)
Age (years)	27.29 (7.27)	27.22 (6.93)	27.43 (8.05)
Height (cm)	174.70 (9.89)	178.66 (7.33)	165.77 (9.08)
Weight (kg)	86.04 (22.74)	87.98 (22.64)	81.69 (22.52)
BMI (kg/m^2^)	28.19 (7.20)	27.54 (6.90)	29.66 (7.67)
	*N* (%)	*N* (%)	*N* (%)
Race			
White	164 (74.2%)	119 (77.8%)	45 (66.2%)
Black or African American	11 (5%)	6 (3.9 (%)	5 (7.4%)
Asian	20 (9%)	12 (7.8%)	8 (11.8%)
American Indian or Alaska Native	7 (7%)	4 (2.6%)	3 (4.4%)
Bi-Racial or Mixed Race	4 (1.8%)	2 (1.3%)	2 (2.9%)
Unknown	15 (6.8%)	10 (6.5%)	5 (7.4%)
Ethnicity			
Not Hispanic/Latino	170 (76.9%)	117 (76.5%)	53 (77.9%)
Mexican, Mexican American, Chicano	33 (14.9%)	24 (15.7%)	9 (13.2%)
Puerto Rican	4 (1.8%)	3 (2.0)	1 (1.5%)
Cuban	6 (2.7%)	4 (2.6%)	2 (2.9%)
Other	8 (3.6%)	5 3.3%)	3 (4.4%)
Age Group			
18−24	90 (40.7%)	61 (39.9%)	29 (42.6%)
25−34	101 (45.7%)	72 (47.1%)	29 (42.6%)
35−44	25 (11.3)	17 (11.1%)	8 (11.8%)
45−54	2 (0.9%)	2 (1.3%)	0 (0%)
55−64	3 (1.4%)	1 (0.7%)	2 (2.9%)
Occupation			
Administrative Support	9 (4.1%)	6 (3.9%)	3 (4.4%)
Athlete	2 (0.9%)	1 (.7%)	1 (1.5%)
Civil Service	5 (2.3%)	5 (3.3%)	0 (0%)
Craftsman/Trade/Laborer/Contractor	8 (3.6%)	7 (4.6%)	1 (1.5%)
Official/Management	9 (4.1%)	6 (3.9%)	3 (4.4%)
Professional	42 (19.0%)	23 (15.0%)	19 (27.9%)
Sales	15 (6.8%)	11 (7.2%)	4 (5.9%)
Student	67 (30.3)	48 (31.4%)	19 (27.9%)
Technician	16 (7.2%)	11 (7.2%)	5 (7.4%)
Technology	15 (6.8%)	15 (9.8%)	0 (0%)
Content Creator/Streamer	4 (1.8%)	2 (1.3%)	2 (2.9%)
Unemployed	9 (4.1%)	5 (3.3%)	4 (5.9%)
Other	20 (9.0%)	13 (8.5%)	7 (10.3%)
Handedness			
Right	196 (88.7%)	135 (88.2%)	61 (89.7%)
Left	17 (7.7%)	11 (7.2%)	6 (8.8%)
Ambidextrous	8 (3.6%)	7 (4.6%)	1 (1.5%)
VGP Level			
Casual	145 (65.6%)	91 (59.5%)	54 (79%)
Amateur	50 (22.6%)	39 (25.5%)	11 (16.2%)
Semi-professional	24 (10.9%)	21 (13.7%)	3 (4.4%)
Professional	2 (0.9%)	2 (1.3%)	0 (0%)

### Design and instrumentation

This study's design was a quantitative, cross-sectional, non-experimental survey study aimed to be exploratory and descriptive in nature. The survey was designed and administered online using Qualtrics and was composed of four sections: (1) Video Game Behaviors, (2) Physical Activity Behaviors via the long form of the International Physical Activity Questionnaire (IPAQ), (3) Demographics, and (4) Health History Information. The purpose of using survey methodology lies within three primary reasons. First, using an online survey permits for a wider variety of participants in our sample. Second, while current wearable fitness devices are capable of recording objective PA and sedentary behavior measurements, they are either (1) incapable of appropriately calculating and classifying sedentary behavior time or (2) simply measure sedentary behavior as one cumulative estimate (6, 27, 28). These types of measures lack the context of what domains and activities are performed, inhibiting the creation of appropriate sedentary behavior interventions and guidelines. Third, the perceptions an individual provides pertaining to their own PA and sedentary behavior are important, as they can be used to educate or guide the individual in behavior change (28, 29). To mitigate survey fatigue, participants were provided instructions to take breaks as needed. Each participant's respective survey link remained open for two weeks to allow for any duration or frequency of breaks taken.

### Video game behaviors

This portion of the questionnaire was developed specifically for this study. The questions were based on previous surveys created to evaluate game play behaviors of VG and traditional card game players (16, 17, 30). The present questionnaire included 35 customized VG play behavior questions. This section included a combination of single response and open-ended questions. Single response questions included the participant's VGP categorization (casual, amateur, semi-professional, or professional player), and frequencies, in days out of seven, of the participant's total video game play. Fill-in-the-blank questions asked the participant for the durations, in hours and minutes, on a typical day of (1) VG play in total by weekday and by weekend day, (2) VG play by platform [console, PC, Virtual Reality (VR), and arcade] by weekday and by weekend day, and (3) 16 game-mechanic based genres [e.g., action, fighting, Massively Multiplayer Online Role-Playing Game (MMORPG), etc.] by weekday and weekend day.

### International physical activity questionnaire (IPAQ)

The 27-question long form of the IPAQ was administered to evaluate the frequencies (in days) and durations (in hours and minutes) a participant spent engaged in moderate and vigorous physical activities and walking over the last seven days. Frequencies were measured using single-response with responses from 0 to 7, while durations were measured using open-response textboxes. These questions are broken down into five sections, (1) occupational physical activities (OPA), (2) transportation physical activities, (3) housework, house maintenance, and caring for family physical activities, (4) recreation, sport, and leisure time physical activities (LTPA), and (5) time spent sitting. Definitions pertaining to physical activities were provided to the participants, and definitions for moderate and vigorous physical activities and exercise were provided for each question asking for its respective activity.

### Demographics

The demographic section comprised 12 questions asking for the participant's age, height (in feet and inches), weight (in pounds), binary sex, gender, handedness, race, ethnicity, occupation, education, and smartphone ownership. Binary sex, race, and ethnicity questions were influenced by the U.S. 2020 Census questions.

### Health history

The health history questionnaire (HHQ) section was comprised of four subsections of diseases and disorders categories: (1) heart and circulatory, (2) respiratory, (3) musculoskeletal, and (4) psychological. Each subsection contained one single response “Yes or No” question asking if the participant had a diagnosis of a disease or disorder in that subsection. Those who responded “yes” were provided a check list of diseases and disorders to choose from within that subsection, while those who responded “no” were moved onto the next subsection. The check lists for each category of disease and disorder were influenced by HHQs given to patients by clinicians and practices such as Versus Arthritis’ Musculoskeletal Health Questionnaire and the TriHealth Physician Partner's Health History Questionnaire. Psychological conditions listed were in reference to those listed on websites such as the National Institute of Mental Health and National Alliance on Mental Illness.

### Procedure

Before participant recruitment began, approval from the Institutional Review Board (IRB) was obtained. Data collection occurred between December 2023 through June 2024. Participants were recruited via non-probability sampling using convenience, voluntary, and snowball sampling methods. Recruitment methods involved (1) placing flyers with the QR code and link to the survey around the university campus, inside coffee shops, cafes, arcades, breweries, and game shops around a southcentral metroplex area, (2) placing advertisements inside university newsletters and on social media pages, (3) advertising the study using chat boxes within various VGs centered within North American servers [e.g., League of Legends (LoL), Dota 2, Old School Runescape, Final Fantasy XIV], (4) email communication, and (5) attending video game related tournaments and conventions.

Prior to participation, individuals were provided information about the study. If interested, they would either enter or click on a link to the survey or scan a QR code directing them to the study. The survey began with an informed consent form for all participants. If the participant provided consent, they completed the inclusionary criteria questions. Those who answered “No” to any of these questions were unable to participate in the study. If they met the inclusion criteria, the participant completed each of the four sections of the survey (i.e., VG behaviors, IPAQ, Demographics, and Health History). Upon completion, the participant was debriefed, including being thanked for their time and instructed to not discuss the questions or their answers with other potential participants.

### Statistical analysis

Statistical analysis of the data was performed using IBM SPSS Statistics software, Version 29. Descriptive statistics were used to analyze each section of the Qualtrics survey. Only surveys completed to their entirety and in English were kept for data analysis.

Duration responses greater than or equal to 24 h were divided by the respective number of days per weekday or weekend day the participant played (i.e., 24 h/2 weekend days, 24 h/3 weekdays, etc.). To consider for sleep, duration responses greater than 20 h per day were also divided by the respective number of days per weekday or weekend day (i.e., 20 h/4 weekdays). Any duration responses with single digits and no time specification were treated as hours. This is due to the uncommonness and unlikelihood that participants record one to four or six to nine minutes of VG play or PA time. Time responses consisting of factors of five starting at ten were treated as minutes unless specified as hours. Hour ranges that were provided (i.e., 4–6, 2–5, etc.) were averaged out in minutes, and any responses such as “# +” or “# or more hours” were treated as the number given (i.e., 10 + equals 10 h). Weekly durations for PA and VG play were calculated as min/week = (minutes on an average weekday × 5) + (hours on an average weekend day × 2). Results are reported in mean and standard deviation unless otherwise stated.

## Results

### Video game play behaviors

On average, participants played VGs over five days per week. These participants spent an average of 3.41 (SD 2.45) hours playing VGs on a weekday and 4.81 (SD 3.09) hours on a weekend day, resulting in a weekly average of 26.56 (SD 16.58) hours. PC reported the longest duration (17.59 [SD 17.51] hours per week), with the second longest duration occurring on consoles (8.28 [SD 12.06] hours per week). Few participants engaged in VG play using VR or traditional arcade platforms, with durations being 21.67 (SD 123.69) minutes per week and 39.64 (SD 232.84) minutes per week common respectively. Based on play time, the top three most popular genres were Shooters, Action, and Tabletop games. Examples of games in these genres are Halo, Monster Hunter, and Magic the Gathering respectively. Among male participants, the most popular genre were Shooter games (*N* = 122), while Simulation games were the most popular among female participants (*N* = 38). See Tables 2, 3 for additional details on durations of VG play by age group, genre, and platform.

**Table 2:** Frequency and duration of video game play by week, day, console and age group.

	Total (*N* = 221)		Male (*N* = 153)		Female (*N* = 68)	
	*N*	Mean (SD)	*N*	Mean (SD)	*N*	Mean (SD)
Total time spent (min)						
Week	218	1593.45 (994.88)	151	1655.79 (953.88)	67	1452.94 (1,075.84)
Weekend day	218	288.31 (185.70)	153	302.42 (180.92)	67	256.51 (193.68)
Weekday	221	204.41 (146.71)	153	211.76 (138.66)	68	187.87 (163.27)
Frequency of play (d/wk)	221	5.29	153	6.11	68	3.43
Time spent by console (min/wk)						
Personal Computer (PC)	220	1,055.37 (1,050.34)	152	1,123.82 (1,027.73)	66	897.73 (1,092.31)
Console	218	496.93 (723.79)	152	490.79 (788.03)	68	510.66 (559.02)
Arcade	221	39.64 (232.84)	153	30.98 (228.87)	68	59.12 (242.12)
Virtual reality (VR)	221	21.67 (123.69)	153	23.86 (130.45)	68	16.76 (107.65)
Time spent by age group (min/wk)						
18−24	90	1,635.90 (926.21)	60	1,753.25 (886.21)	29	1,393.10 (975.04)
25−34	99	1,580.73 (999.80)	71	1,674.15 (1,005.01)	28	1,343.82 (963.52)
35−44	25	1,693.80 (1,218.09)	17	1,426.76 (948.13)	8	2,261.25 (1,578.35)
45−54	2	555.00 (190.92)	2	555.00 (190.92)	0	
55–64	3	610.00 (315.12)	1	600.00	2	615.00 (445.48)

**Table 3:** Weekly duration of video game play and leisure time physical activity by video game genre.

	Total (*N* = 221)		Male (*N* = 153)		Female (*N* = 68)	
	*N*	Mean (SD)	*N*	Mean (SD)	*N*	Mean (SD)
Time spent playing VGs by genre per week (min/wk) followed by time spent in LTPA per week (min/week)						
Sandbox	3	2,060 (341.17)	2	2,010.00 (466.69)	1	2,160
		0				
MMORPG	42	1,089.76 (841.76)	29	1,220.34 (871.70)	13	798.46 (717.45)
		127.38 (201.48)				
Shooter	122	1,025.98 (729.10)	96	1,026.30 (707.56)	26	1,024.81 (818.80)
		158.67 (256.05)				
RPG	24	839.79 (847.19)	16	637.81 (502.17)	8	1,243.75 (1,240.21)
		145.21 (318.04)				
MOBA	48	831.15 (759.65)	37	836.35 (642.61)	11	813.64 (1,106.87)
		118.85 (231.45)				
Tabletop	75	814.93 (731.86)	51	800.98 (640.28)	24	844.58 (911.43)
		148.00 (235.99)				
Simulation	57	802.54 (752.89)	19	921.05 (765.87)	38	743.29 (749.51)
		144.82 (216.41)				
Action	103	795.73 (641.76)	71	900.21 (687.39)	32	563.91 (455.77)
		139.34 (220.71)				
Strategy	43	791.05 (786.72)	33	716.82 (650.72)	10	1,036.00 (1,138.37)
		99.07 (157.43)				
Survival	8	787.50 (412.82)	4	735.00 (284.08)	4	840.00 (556.42)
		78.75 (146.04)				
Fighting	54	600.93 (762.99)	42	678.21 (834.92)	12	330.42 (322.52)
		147.18 (205.11)				
Other	9	587.22 (625.31)	6	425.83 (620.37)	3	910.00 (606.22)
		218.33 (232.03)				
Adventure	3	555.00 (328.98)	1	315.00	2	675.00 (360.62)
		50.00 (86.60)				
Sports & Racing	28	526.61 (360.36)	23	571.96 (358.30)	5	318.00 (322.83)
		153.66 (221.24)				
Rogue-like	54	507.50 (400.14)	39	495.13 (388.78)	15	539.67 (440.85)
		157.04 (207.21)				
Puzzle	6	461.67 (269.57)	2	525.00 (190.92)	4	362.50 (311.81)
		285.00 (198.09)				

In order from longest to shortest genre played.

The numbers below each weekly VG play time is the average amount of LTPA each genre spent per week in minutes.

### Physical activity behaviors

When observing the total amount of time adult VGPs spent engaged in MVPA (including moderate and vigorous occupational, transportation, in-home, yardwork, and LTPA), participants reported 15.43 (SD 16.79) hours per week. The majority of this time was spent engaged in occupation-related PA (5.11 [SD 8.70] hours per week). Leisure time MVPA was the second least reported, with participants spending only 2.39 (SD 3.72) hours per week. See Table 4 for additional details on PA by age group and domain and for sedentary duration.

**Table 4:** Duration of moderate to vigorous physical activity and sedentary behavior.

	Total (*N* = 221)		Male (*N* = 153)		Female (*N* = 68)	
	*N*	Mean (SD)	*N*	Mean (SD)	*N*	Mean (SD)
Time spent in MVPA by domain (min/wk)						
Total MVPA	221	925.56 (1,007.47)	153	829.71 (820.25)	68	1,141.21 (1,318.43)
Leisure	221	143.52 (222.96)	153	148.71 (233.84)	68	131.84 (197.43)
Occupational	221	306.60 (522.13)	153	294.80 (510.48)	68	333.12 (550.40)
Yard	221	286.25 (443.95)	153	243.66 (317.64)	68	382.07 (636.26)
In Home	221	189.91 (321.10)	153	142.54 (190.96)	68	294.18 (489.57)
Time spent in LTPA by age group (min/wk)						
18–24	90	161.36 (214.66)	61	177.83 (223.95)	29	126.72 (192.76)
25–34	101	116.98 (199.94)	72	115.62 (204.91)	29	120.34 (190.49)
35–44	25	163.80 (322.50)	17	170.29 (357.73)	8	150.00 (252.53)
45–54	2	342.40 (194.45)	2	342.40 (194.45)	0	0.00
55–64	3	200.00 (229.13)	1	0.00	2	300.00 (212.13)
Sedentary time (min)						
Week	221	3,068.44 (1,488.60)	153	3,172.19 (1,552.35)	68	2,835.00 (1,315.05)
Weekend Day	221	428.48 (229.09)	153	443.82 (237.15)	68	393.97 (207.322)
Weekday	221	442.29 (227.21)	153	456.91 (236.37)	68	409.41 (202.925)

### Chronic health conditions

Among the participants, 98 reported having at least one CHC, with 56 being male and 42 being female. Of these participants, 75 reported only having one CHC. Within the Heart and Circulatory conditions category, the most reported conditions were myocardial infarction, heart failure, and hypertension, while asthma was the most common within the Respiratory condition category. The most common Musculoskeletal condition was a herniated disc. Finally, the most common psychological conditions were anxiety disorders, depression, and attention deficit/hyperactivity disorder (ADHD). See Table 5 for additional details pertaining to CHC information.

**Table 5:** Frequencies of chronic health conditions.

	Total (*N* = 221)	Male (*N* = 153)	Female (*N* = 68)
	*N* (%)	*N* (%)	*N* (%)
Yes vs. no for any CHC			
Yes	98 (44.3%)	56 (36.6%)	42 (61.8%)
No	123 (55.7%)	97 (63.4%)	26 (38.2%)
CHC category			
Heart & circulatory	8 (3.6%)	6 (3.9%)	2 (2.9%)
Respiratory	34 (15.4%)	21 (13.7%)	13 (19.1%)
Musculoskeletal	6 (2.7%)	3 (2.0%)	3 (4.4%)
Psychological	77 (34.8)	38 (24.8%)	39 (57.4%)
Number of CHC categories			
1	75 (33.9%)	47 (30.7%)	28 (41.2%)
2	19 (8.6)	6 (3.9%)	13 (19.1%)
3	4 (1.8%)	3 (2.0%)	1 (1.5%)

## Discussion

This cross-sectional study among adult VGPs in the U.S. describes the differences in VG play and PA behaviors, investigating the need for more effective PA interventions and sedentary behavior guidelines for this population. In this study, adult VGPs reported an average of 51.14 (SD 24.81) hours per week sitting while at work, at home, while doing course work, and during leisure time. The current participants reported spending approximately 8.65 h longer in weekly sedentary behavior duration than the participants of a study conducted in early 2024 (approximately 42.49 h per week) (31). Though our results seem inconsistent to this study, Dowdell and colleagues’ population mainly consisted of a higher proportion of esports athletes (*N* = 304) compared to casual players (*N* = 228). In comparison, the present study's sample consisted of a higher proportion of casual players (*N* = 195) compared to professional players (*N* = 26). The esports athletes of Dowdell and colleagues’ study were reported to be less sedentary (31 h per week) compared to casual VGPs (53.98 h per week). Therefore, our results of weekly sedentary behavior duration are fairly congruent with those of Dowdell et al. (31). When comparing the current study's results to sitting behavior reported by U.S. adults who are not specifically VGPs, the participants in this study reported slightly higher duration averages. Compared to data collected in 2019, the present study's participants spent an average of 7.31 h per day sitting compared to 7.1 h per day reported using a single-question questionnaire (32). However, Matthews and colleagues’ (2019) sample was older on average (45.3 years) and had a relatively even ratio of males to females (49% and 51%, respectively). Additionally, when Matthews and colleagues (2019) used a 24-hour recall, their participants reported an average of 9.5 h per day. In comparison to Arnaez et al.'s (17) results, sitting results reported by participants in this study were lower than those reported by the VG and tabletop players from 2018. Participants from Arnaez and colleagues (17) reported 9.26 h per any given day (64.84 h per week) of sedentary time. Although these results were higher than what were found in the present study, this can be attributed to the fact Arnaez et al.'s sample primarily consisted of tabletop players. Due to the nature and rules of play, tabletop game matches typically last 60 min or more. This would explain why the average sedentary time is higher compared to the present study's sample. These results suggest several things. First, the present study's participants may not accurately recall their sedentary behaviors and durations. Second, the genre of VG play matters in the context of sedentary behavior cumulation. Third, the lengthy durations of sitting time give an insight to how sedentary behavior can quickly compound over the course of a week. This is a concern especially for populations who tend to partake in sedentary hobbies like VGs. For instance, the top two occupations reported by the participants were students (*N* = 67) and professional occupations, including those in technology (*N* = 57). These two options require sedentary behavior to perform tasks (i.e., studying for school, work-related computer use, etc.). If participants are predominantly sedentary during the workday and engage in sedentary VG play afterward, then the amount of time spent sedentary will compound. As a result, this accumulation of sedentary time will increase the risk of developing physical health risks in this population.

Based on our results, over half (26.56 h) of the participants’ weekly sitting time stemmed from playing VGs. The averages for VG play per week were substantially more than previous responses from similar studies observing VG play times in adult VGPs (17). In 2018, participants reported spending 12.6 h per week playing, showing a 14-hour increase over the last six years. When separated into weekday and weekend day averages, participants from this study played VGs approximately 3.41 h and 4.81 h a day respectively, resulting in an average of 3.79 h of play time on any given day. Compared to 2018 (17), these numbers increased by 1.71 h per weekday, 2.71 h per weekend day, and by 1.99 h on any given day, respectively. Assessing the differences between our results and those of Matthews et al. (2019), the latter reported 3.5 h of leisure time sedentary behavior stemmed from the combination of TV/video and internet/computer use, which was only a 0.29-hour difference (32). However, Matthews and colleagues (2019) did not evaluate solely VG play, which may have been included in their participants’ TV/video and or internet/computer use responses (i.e., electronic media or screen-based behaviors). Since these activities were not exclusively listed as VG play, we cannot make a direct comparison about VG play. Regardless, our results are consistent with Matthews et al.'s (2019) findings that leisure time sedentary activities account for the majority of sedentary behavior duration (32). These findings all suggest that these increases in VG play time are likely to continue to increase over time, especially considering the 74.6% increase in VG play time over the COVID-19 pandemic (18).

In contrast to VG play behaviors, cumulative MVPA behaviors among the present study's participants averaged 15.23 h per week. When compared to Arnaez et al.'s (2018) results of a mean of 5.2 h per week of MVPA, our results were approximately 10 h higher (17). In comparison to Dowdell and colleague's (2024) MVPA results, the present sample reported approximately 2.36 h less per week, which is relatively consistent with the most recent literature (31). The present study's participants’ average exceeds the current guidelines of at least 2.5 h of MVPA per week (6, 9, 22, 27). When observing PA durations by domain, the longest duration stemmed from occupational activities, with participants reporting an average of 5.11 (SD 8.70) hours per week. LTPA was reported to be the second least engaged domain of PA, amounting to a mean of 2.39 (SD 3.72) hours of MVPA per week. While the participants are meeting the PA guidelines of MVPA cumulatively, we mainly attribute this to the large standard deviations in MVPA by domain, especially in OPA (see Table 4).

Of the four domains of MVPA, OPA had the longest duration and largest standard deviation. This is due to some participants working trade-labor careers such as landscapers or HVAC workers. As such, the lowest reported weekly OPA was zero minutes while the highest reported was 48 h. To date, the current U.S. PA Guidelines and WHO PA guidelines state some or any PA is better than none (6, 9). However, research on OPA has shown these activities provide either no or detrimental effects on physical health (23, 33, 34). As hypothesized by Holtermann et al. (2018), this may be due to OPA being too low intensity, too long in duration, and performed in awkward positions with insufficient recovery times (35). Furthermore, current literature supports that LTPA is optimal to improve physical health and reduce the development of CHCs or all-cause mortality (33, 35–37). This is particularly apparent when LTPA is compared to OPA and may explain why public health and epidemiological research focuses on LTPA (32, 37, 38). These insights may explain three things. First, the long durations and standard deviations of OPA in the present study's participants may skew the total weekly duration of MVPA. Second, the skew in total MVPA may explain why the participants were found to meet the PAG. Third, the present study's participants are, realistically, likely not meeting the PAG due to the little to no health benefits provided by OPA. Considering this, when strictly examining our participants’ LTPA, our results are concurrent with literature addressing U.S. adults not meeting the current recommended PA guidelines (9, 22, 25). Such information emphasizes why all domains of PA should be investigated. Furthermore, this particular data suggests U.S. adult VGPs may be more at risk for CHCs due to sedentary habits established during the COVID-19 pandemic. In a review examining the effects of the pandemic on PA, it was found that the pandemic had a statistically significant negative effect on PA and statistically significant increases in time spent sitting, engaged in screen time (including VGs), and total sedentary behavior (39). These changes in duration of sedentary and PA times are likely due to social distancing practices where many individuals were required to forgo in-person PA (e.g., going to the gym or outdoor spaces, traveling to work, etc.). However, there are other potential variables that may have had an impact on the durations of VG play and PA investigated by this study such as VG mechanic genres.

The first game-play mechanic that may influence VG play duration is time limits, constraints, and minimums. According to our descriptives, those who played genres relying on one of these mechanisms typically reported increased durations of LTPA per week and lower times of VG play when compared to the genres that do not. These increased durations of LTPA and lower times of VG play may be due to this group of players having the opportunity to quit or pause the game. The cue to quit or pause a game will signal that a time limit has been reached or that the round is over, prompting the player to decide whether to take a break or continue playing. In contrast, players of genres without time limits will not have cues to indicate when to stop the game. However, there is little literature discussing the relationship between game time limits and PA durations. The second game-play mechanic that may influence PA and VG behavior is whether or not there is a narrative or storytelling element to the game. When observing genres that may rely on either of these mechanics, despite the sample sizes being very small, participants reported longer durations of VG play per week and shorter durations of PA. One possible explanation for this occurrence is how immersed or transported the player is into the world and the story of the VG they are playing. As Green and Brock (2000) suggest, when an individual is transported while engaging in an activity (e.g., VGs), there are increases in affect and focus (40). These increases may result in a lack of awareness of time and the real world, potentially increasing VG play. In turn, this means the player will have less time to engage in PA since it is already being spent immersed in the game. Finally, a third game-play mechanic potentially influencing VG and PA behavior is multi-player capability or requirements. Some genres require multiple players to be able to play the game while others do not. Additionally, some games may not require multiple players to function but offer the option to play with others. By having the opportunity to play VGs with multiple people, VGPs are likely drawn to the social appeal of playing VGs with others. Socialization is enjoyable for most individuals, so when socialization is coupled with another pleasurable activity, individuals are likely to engage in that activity more frequently and for longer durations (41). Exploring specific VG genres and durations of VG platforms may be beneficial to improve the physical health of adult VGPs. Therefore, researchers and practitioners may be able to develop specific guidelines and interventions by VG genre and platform.

Finally, considering participants number of CHCs, we observed that those with two or three CHCs engaged in longer durations of LTPA per week (3.35 h, and 2.58 h respectively) than those without a CHC (2.40 h). Surprisingly, those who reported one CHC engaged in the least amount of weekly LTPA (2.12 h). We hypothesize two reasons for the increase in LTPA between having one CHC compared to having two or three CHCs. First, it is likely those with two or more CHCs have been made aware of their ailments by a health care provider. The provider is probably suggesting their patient engage in LTPA to mitigate the effects of the respective CHCs, explaining the increase in weekly duration. The second reason considers the participants’ awareness of the severity of the situation. Self-awareness of problems or faults often incites change in oneself, which may explain these participants’ approach to managing their situation by performing LTPA. Additionally, a participant being aware of having two or more CHCs may provide a sense of urgency to engage in these changes. In comparison, those with one CHC may not have the sense of urgency or sense of severity of CHC to encourage behavior change.

### Strengths, limitations, and future research

To the best of our knowledge, this study is the most updated descriptive data on adult VGPs, VG play behaviors, and VGP PA engagement. Additionally, this study is one of the first to assess the time VGPs spend playing various VG genres, with the most recent prior studies being Weaver et al. (30) and Difrancisco-Donoghue et al. (18). Comparatively, we observed sixteen genres compared to the previous studies’ three and eight genres, respectively. As with all studies, this one is not without limitations. First, due to the nature of self-report survey studies, various biases were inevitable. Primary biases include sampling, non-response, and self-selection biases. Other biases associated with self-report studies include social response, recall, and non-differentiation bias. Such biases may have resulted in an overestimation of PA engagement and an underestimation of VG play time and sedentary time. Overestimations of PA may be due to participant desire to avoid being judged against a perceived societal expectation, while underestimations of VG play and sedentary time may be due to the same reason or a lack of awareness of real-world time. Future research should consider using objective methods to measure the frequencies and durations of PA and VG play. Regarding sedentary behavior, a variety of approaches to measuring sedentary activities and domains should be contemplated. First, potential studies investigating sedentary behavior should consider breaking out each domain into specific activities and specifying what platforms they took place on. Specifically, current literature typically groups electronic activities into one group such as “electronic media” or “screen-based” behaviors. These groups should be further broken into detailed activities such as VG play, social media use, watching movies, and watching tv shows. Providing more detailed information about each activity and what platforms are being used would enable researchers to develop more informed guidelines and better interventions for each leisure time sedentary activity. Examples of such interventions include incorporating screen time-limits into electronic activities or considering the creation of health warning labels detailing the impact of prolonged sedentary behavior on one's physical and mental health. If subjective measurement of sedentary behavior frequencies, durations, and activities is utilized, researchers should consider using a 24-hour recall. The second limitation is our sample comprised adult VGPs within the U.S. and could not include those without convenient internet access or those unable to read English. These populations can consist of lower-income groups who cannot access the internet to take our survey and those incapable of confidently reading English. Third, our sample size was small and could have influenced the results of our statistical analysis. However, comparable studies by Arnaez et al. (17) and Ballard et al. (16) also had limited sample sizes of 292 and 116, respectively. Future research should consider using a larger sample size to alleviate these limitations. Fourth, we were only able to enroll a very small number of semi-professional (*N* = 24) and professional (*N* = 2) VGPs. Potential studies should obtain wider access to semi-professional and professional esports teams (i.e., semi-professional collegiate esports teams and professional esports organizations such as Team SoloMid, Moist Esports, OpTic Gaming, etc.). Finally, due to the non-experimental design of this study we cannot draw conclusions about causality.

## Conclusion

The purpose of this study was to describe VG play and PA behaviors in adult VGPs. The results of the current study found that adult VGPs spend 26.56 h per week playing video games. This was a 14-hour increase in VG play per week from 2018 (17). Comparatively, participants reported spending a mean of 2.39 h of LTPA per week. When observing weekly VG play by genre, the most popular and longest played genre was Shooter games. The second and third most played genres were Action and Tabletop games respectively. Approximately 44% of participants had at least one CHC, and those who reported two or three CHCs engaged in more weekly LTPA than those without. However, participants who reported one CHC engaged in the least amount of weekly LTPA. The current study's results may benefit practitioners, clinicians, and researchers alike in developing novel interventions and guidelines for PA and sedentary behavior in this population. Regardless, continued research on VG play and PA behaviors in adult VGPs is needed to improve this population's physical health.

## Data Availability

The raw data supporting the conclusions of this article will be made available by the authors, without undue reservation.
